# Supplementary material for: Single Cell Transcriptomic and Chromatin Profiles Suggest Layer Vb Is the Only Layer With Shared Excitatory Cell Types in the Medial and Lateral Entorhinal Cortex
Source: Front Neural Circuits. 2022 Jan 26;15:806154. doi: 10.3389/fncir.2021.806154 (PMC8826650; doi:10.3389/fncir.2021.806154)
Supplement: Supplementary file 14 [file Data_Sheet_1.ZIP › 9/knownResults.html]

9 - Homer Known Motif Enrichment Results


# Homer Known Motif Enrichment Results (9)

Homer *de novo* Motif Results  
Gene Ontology Enrichment Results  
Known Motif Enrichment Results (txt file)  
Total Target Sequences = 15748, Total Background Sequences = 33798

|  |  |  |  |  |  |  |  |  |  |  |  |
| --- | --- | --- | --- | --- | --- | --- | --- | --- | --- | --- | --- |
| Rank | Motif | Name | P-value | log P-pvalue | q-value (Benjamini) | # Target Sequences with Motif | % of Targets Sequences with Motif | # Background Sequences with Motif | % of Background Sequences with Motif | Motif File | SVG |
| 1 | T G C A A G C T C T G A A T C G G A C T C T A G G T A C G A T C G T C A A G T C G T A C G A C T C T A G A T C G G C A T C A T G C A T G G A T C G T A C C T G A | CTCF(Zf)/CD4+-CTCF-ChIP-Seq(Barski\_et\_al.)/Homer | 1e-311 | -7.171e+02 | 0.0000 | 1263.0 | 8.02% | 770.6 | 2.28% | motif file (matrix) | svg |
| 2 | A T G C G A T C C G A T A C G T A C G T A C T G C A G T A G C T | Sox3(HMG)/NPC-Sox3-ChIP-Seq(GSE33059)/Homer | 1e-303 | -6.987e+02 | 0.0000 | 5486.0 | 34.83% | 7388.8 | 21.85% | motif file (matrix) | svg |
| 3 | A T G C A G T C G C A T A G C T A C G T T C A G C G A T A G C T G A T C A T C G | Sox10(HMG)/SciaticNerve-Sox3-ChIP-Seq(GSE35132)/Homer | 1e-303 | -6.985e+02 | 0.0000 | 5304.0 | 33.68% | 7057.9 | 20.87% | motif file (matrix) | svg |
| 4 | A T G C G A T C C G T A A G C T C A G T A T C G G C A T A G C T G A C T A C T G | Sox17(HMG)/Endoderm-Sox17-ChIP-Seq(GSE61475)/Homer | 1e-284 | -6.552e+02 | 0.0000 | 2740.0 | 17.40% | 2839.4 | 8.40% | motif file (matrix) | svg |
| 5 | A T G C A G T C G A T C C G T A A C G T A C G T A C T G A C G T A G C T G A T C | Sox2(HMG)/mES-Sox2-ChIP-Seq(GSE11431)/Homer | 1e-283 | -6.530e+02 | 0.0000 | 3331.0 | 21.15% | 3773.7 | 11.16% | motif file (matrix) | svg |
| 6 | A T G C G T A C C G T A A G C T G C A T T A C G A G C T A G C T A G T C A G C T | Sox6(HMG)/Myotubes-Sox6-ChIP-Seq(GSE32627)/Homer | 1e-274 | -6.316e+02 | 0.0000 | 4951.0 | 31.44% | 6606.5 | 19.53% | motif file (matrix) | svg |
| 7 | C T G A T C G A C G T A A T G C C G T A C G T A C G A T C T A G T C A G G A T C | Sox15(HMG)/CPA-Sox15-ChIP-Seq(GSE62909)/Homer | 1e-262 | -6.050e+02 | 0.0000 | 3685.0 | 23.40% | 4466.5 | 13.21% | motif file (matrix) | svg |
| 8 | A C G T A G T C A G T C C G A T A C G T A C G T A C T G A C G T A T G C G A C T A C T G T A C G | Sox21(HMG)/ESC-SOX21-ChIP-Seq(GSE110505)/Homer | 1e-252 | -5.821e+02 | 0.0000 | 5587.0 | 35.48% | 7925.1 | 23.43% | motif file (matrix) | svg |
| 9 | A G T C A G T C C G A T A C G T A C G T A C T G A C G T A G C T A G T C A G T C | Sox4(HMG)/proB-Sox4-ChIP-Seq(GSE50066)/Homer | 1e-250 | -5.759e+02 | 0.0000 | 3138.0 | 19.93% | 3628.7 | 10.73% | motif file (matrix) | svg |
| 10 | C G T A C T A G T C A G T C A G A G T C A T G C A G T C G C A T A G C T A C G T A T C G C G A T | Sox9(HMG)/Limb-SOX9-ChIP-Seq(GSE73225)/Homer | 1e-243 | -5.604e+02 | 0.0000 | 3227.0 | 20.49% | 3810.0 | 11.27% | motif file (matrix) | svg |
| 11 | T A G C A G T C T G A C A G T C C T A G A T C G A G T C C A T G T G A C A G T C G T A C A G T C A G T C G C A T C T A G A T C G G C A T A C T G A T C G G A T C | BORIS(Zf)/K562-CTCFL-ChIP-Seq(GSE32465)/Homer | 1e-190 | -4.394e+02 | 0.0000 | 1560.0 | 9.91% | 1470.8 | 4.35% | motif file (matrix) | svg |
| 12 | T G A C T A G C T C A G T C G A T C G A C G T A A G T C C G T A C G T A C G A T C T A G T A C G | Sox7(HMG)/ESC-Sox7-ChIP-Seq(GSE133899)/Homer | 1e-169 | -3.892e+02 | 0.0000 | 1316.0 | 8.36% | 1206.1 | 3.57% | motif file (matrix) | svg |
| 13 | A G T C G A C T C A G T A C T G C T A G T G A C G C T A A T G C G C A T A T C G C G A T A C T G G A T C G T A C G T C A C T G A | NF1(CTF)/LNCAP-NF1-ChIP-Seq(Unpublished)/Homer | 1e-95 | -2.203e+02 | 0.0000 | 1562.0 | 9.92% | 1926.6 | 5.70% | motif file (matrix) | svg |
| 14 | T A G C C T A G T C G A G A C T A C T G C T G A A G T C T C A G G C A T T G A C C T G A A G C T | Atf7(bZIP)/3T3L1-Atf7-ChIP-Seq(GSE56872)/Homer | 1e-80 | -1.862e+02 | 0.0000 | 1427.0 | 9.06% | 1799.7 | 5.32% | motif file (matrix) | svg |
| 15 | T C G A G C A T A C T G C T G A A G T C T C A G G A C T G T A C C G T A A G C T A G T C G A T C | c-Jun-CRE(bZIP)/K562-cJun-ChIP-Seq(GSE31477)/Homer | 1e-80 | -1.860e+02 | 0.0000 | 1013.0 | 6.43% | 1135.0 | 3.36% | motif file (matrix) | svg |
| 16 | T G C A T C G A T C G A A C G T C A T G C G T A A G T C T C A G A C G T G T A C C G T A A G C T | CREB5(bZIP)/LNCaP-CREB5.V5-ChIP-Seq(GSE137775)/Homer | 1e-80 | -1.850e+02 | 0.0000 | 1100.0 | 6.98% | 1273.7 | 3.77% | motif file (matrix) | svg |
| 17 | A T G C T C A G T C G A G C A T A C T G C G T A A G T C T C A G G A C T T G A C C G T A A G C T | Atf2(bZIP)/3T3L1-Atf2-ChIP-Seq(GSE56872)/Homer | 1e-76 | -1.770e+02 | 0.0000 | 1097.0 | 6.97% | 1289.0 | 3.81% | motif file (matrix) | svg |
| 18 | C G A T T G C A T G C A G A T C C G T A A C T G T G A C G A C T C A T G A C T G | Tcf21(bHLH)/ArterySmoothMuscle-Tcf21-ChIP-Seq(GSE61369)/Homer | 1e-72 | -1.659e+02 | 0.0000 | 3154.0 | 20.03% | 4976.8 | 14.72% | motif file (matrix) | svg |
| 19 | A G C T A C G T A C T G A T G C A G T C C G T A C T G A T A C G | NF1-halfsite(CTF)/LNCaP-NF1-ChIP-Seq(Unpublished)/Homer | 1e-69 | -1.609e+02 | 0.0000 | 4973.0 | 31.58% | 8550.4 | 25.28% | motif file (matrix) | svg |
| 20 | T A C G T C G A G A C T A C T G C T G A A G T C T C A G G A C T T G A C C T G A | Atf1(bZIP)/K562-ATF1-ChIP-Seq(GSE31477)/Homer | 1e-68 | -1.577e+02 | 0.0000 | 1819.0 | 11.55% | 2565.5 | 7.59% | motif file (matrix) | svg |
| 21 | G T C A T G C A G C T A A G T C C G T A A C T G T G A C G C A T T C A G C A G T | Ap4(bHLH)/AML-Tfap4-ChIP-Seq(GSE45738)/Homer | 1e-62 | -1.445e+02 | 0.0000 | 3812.0 | 24.20% | 6359.0 | 18.80% | motif file (matrix) | svg |
| 22 | T C G A A C G T A C G T C T G A G A T C T C A G G A C T G T C A C G T A A G C T G T C A C T A G A G C T A C G T T C G A | NFIL3(bZIP)/HepG2-NFIL3-ChIP-Seq(Encode)/Homer | 1e-52 | -1.213e+02 | 0.0000 | 1413.0 | 8.97% | 1990.1 | 5.88% | motif file (matrix) | svg |
| 23 | T C G A A G T C C G T A A T C G A T G C C G A T A C T G A G T C A G C T A C T G | Tcf12(bHLH)/GM12878-Tcf12-ChIP-Seq(GSE32465)/Homer | 1e-51 | -1.196e+02 | 0.0000 | 3177.0 | 20.17% | 5278.2 | 15.61% | motif file (matrix) | svg |
| 24 | A G T C C G A T A C T G A T C G T G A C G C T A C A T G A T C G T G A C C G A T A C T G T A G C G T A C G T C A | Tlx?(NR)/NPC-H3K4me1-ChIP-Seq(GSE16256)/Homer | 1e-51 | -1.181e+02 | 0.0000 | 1465.0 | 9.30% | 2094.1 | 6.19% | motif file (matrix) | svg |
| 25 | T C G A A C G T A C T G C T G A A G T C T C A G A G C T G T A C C G T A A G C T G A T C T C G A | JunD(bZIP)/K562-JunD-ChIP-Seq/Homer | 1e-50 | -1.155e+02 | 0.0000 | 408.0 | 2.59% | 379.1 | 1.12% | motif file (matrix) | svg |
| 26 | T C A G A C G T T C G A T A G C A G T C C G T A A C T G G T A C A C G T A C T G A T C G A G T C | Atoh1(bHLH)/Cerebellum-Atoh1-ChIP-Seq(GSE22111)/Homer | 1e-46 | -1.074e+02 | 0.0000 | 3283.0 | 20.85% | 5562.6 | 16.45% | motif file (matrix) | svg |
| 27 | T A C G C T A G A T G C G A T C G T A C A G T C C T A G A G T C A G T C A G T C G T A C A G T C | Sp1(Zf)/Promoter/Homer | 1e-45 | -1.040e+02 | 0.0000 | 1885.0 | 11.97% | 2918.4 | 8.63% | motif file (matrix) | svg |
| 28 | C T G A T C A G C A G T C T A G A C T G C T A G G A T C A T C G A C T G C T G A T C A G G A T C | Sp5(Zf)/mES-Sp5.Flag-ChIP-Seq(GSE72989)/Homer | 1e-45 | -1.037e+02 | 0.0000 | 4212.0 | 26.74% | 7427.5 | 21.96% | motif file (matrix) | svg |
| 29 | A C G T T C G A T C G A A G T C G T C A T A C G A T G C A C G T A C T G A G C T | Myf5(bHLH)/GM-Myf5-ChIP-Seq(GSE24852)/Homer | 1e-44 | -1.026e+02 | 0.0000 | 2256.0 | 14.32% | 3618.9 | 10.70% | motif file (matrix) | svg |
| 30 | C T A G T C G A T G A C A G T C C G T A A C T G G T A C A C G T A C T G A C T G | BHLHA15(bHLH)/NIH3T3-BHLHB8.HA-ChIP-Seq(GSE119782)/Homer | 1e-44 | -1.016e+02 | 0.0000 | 4030.0 | 25.59% | 7079.6 | 20.93% | motif file (matrix) | svg |
| 31 | A T G C A T C G T A C G A G C T A T C G C T G A A G T C C T A G A G C T A T G C C T G A A T G C | CRE(bZIP)/Promoter/Homer | 1e-40 | -9.420e+01 | 0.0000 | 879.0 | 5.58% | 1167.3 | 3.45% | motif file (matrix) | svg |
| 32 | T C G A T G A C G T A C C G T A C A G T T G A C A C G T A C T G A G C T A G C T | NeuroG2(bHLH)/Fibroblast-NeuroG2-ChIP-Seq(GSE75910)/Homer | 1e-38 | -8.891e+01 | 0.0000 | 4195.0 | 26.64% | 7511.9 | 22.21% | motif file (matrix) | svg |
| 33 | T C G A T C G A A G T C C G T A C T A G T A G C A C G T A C T G | MyoG(bHLH)/C2C12-MyoG-ChIP-Seq(GSE36024)/Homer | 1e-36 | -8.470e+01 | 0.0000 | 3289.0 | 20.88% | 5736.7 | 16.96% | motif file (matrix) | svg |
| 34 | T G A C A G T C C G T A A C T G G T A C A C G T A C T G A C G T G A C T G A T C | Twist2(bHLH)/Myoblast-Twist2.Ty1-ChIP-Seq(GSE127998)/Homer | 1e-36 | -8.364e+01 | 0.0000 | 4892.0 | 31.06% | 8972.4 | 26.53% | motif file (matrix) | svg |
| 35 | T C A G A C G T A G T C T C G A A G T C T C A G G C A T C T A G C T A G A G C T | Usf2(bHLH)/C2C12-Usf2-ChIP-Seq(GSE36030)/Homer | 1e-35 | -8.144e+01 | 0.0000 | 965.0 | 6.13% | 1361.7 | 4.03% | motif file (matrix) | svg |
| 36 | T C A G T G A C G T A C C G T A A C G T T G A C A C G T T C A G A G C T G A C T | NeuroD1(bHLH)/Islet-NeuroD1-ChIP-Seq(GSE30298)/Homer | 1e-34 | -7.966e+01 | 0.0000 | 2392.0 | 15.19% | 4019.0 | 11.88% | motif file (matrix) | svg |
| 37 | T A C G T G C A A G T C C G T A A C G T T G A C A C G T A C T G A C T G G C A T | TCF4(bHLH)/SHSY5Y-TCF4-ChIP-Seq(GSE96915)/Homer | 1e-32 | -7.596e+01 | 0.0000 | 4206.0 | 26.71% | 7646.0 | 22.61% | motif file (matrix) | svg |
| 38 | T C A G C G T A A G T C A G C T C G T A A G T C C T G A C G T A A G T C G C A T A G T C A G T C A G T C C T G A A C T G T G C A T C G A C A T G A T C G G A T C | Ronin(THAP)/ES-Thap11-ChIP-Seq(GSE51522)/Homer | 1e-30 | -7.083e+01 | 0.0000 | 206.0 | 1.31% | 174.1 | 0.51% | motif file (matrix) | svg |
| 39 | C T G A T C A G A G T C C G T A A T C G A T G C C G A T A C T G A G T C G A C T A T C G A G T C | MyoD(bHLH)/Myotube-MyoD-ChIP-Seq(GSE21614)/Homer | 1e-29 | -6.859e+01 | 0.0000 | 2589.0 | 16.44% | 4482.6 | 13.25% | motif file (matrix) | svg |
| 40 | T C A G A G C T A T G C C G T A A G C T T C A G C A G T A C T G C T G A A G T C | MITF(bHLH)/MastCells-MITF-ChIP-Seq(GSE48085)/Homer | 1e-29 | -6.790e+01 | 0.0000 | 2460.0 | 15.62% | 4235.1 | 12.52% | motif file (matrix) | svg |
| 41 | A G C T T G A C C G A T C G A T C T A G A C G T C A G T C A G T G C T A A G T C | FOXK1(Forkhead)/HEK293-FOXK1-ChIP-Seq(GSE51673)/Homer | 1e-29 | -6.721e+01 | 0.0000 | 2163.0 | 13.73% | 3663.8 | 10.83% | motif file (matrix) | svg |
| 42 | C T G A T C A G G T A C G C T A A C T G T G A C G C A T C A T G | SCL(bHLH)/HPC7-Scl-ChIP-Seq(GSE13511)/Homer | 1e-29 | -6.683e+01 | 0.0000 | 10503.0 | 66.69% | 21094.0 | 62.37% | motif file (matrix) | svg |
| 43 | A G T C C T A G C T A G A G T C G A T C G T A C A G T C C T A G A G T C A G T C A G T C G T A C | Sp2(Zf)/HEK293-Sp2.eGFP-ChIP-Seq(Encode)/Homer | 1e-28 | -6.483e+01 | 0.0000 | 5366.0 | 34.07% | 10136.5 | 29.97% | motif file (matrix) | svg |
| 44 | C G A T C T A G A C G T G T C A C G T A C G T A A G T C C G T A | Foxo3(Forkhead)/U2OS-Foxo3-ChIP-Seq(E-MTAB-2701)/Homer | 1e-28 | -6.461e+01 | 0.0000 | 1573.0 | 9.99% | 2554.0 | 7.55% | motif file (matrix) | svg |
| 45 | G A C T T C A G C T A G A G T C A G T C G T A C A G T C C T G A A G T C A G T C A G T C G A C T A G T C A C T G A T G C | KLF3(Zf)/MEF-Klf3-ChIP-Seq(GSE44748)/Homer | 1e-27 | -6.319e+01 | 0.0000 | 2289.0 | 14.53% | 3937.2 | 11.64% | motif file (matrix) | svg |
| 46 | T C A G C T A G C T A G A C T G A C T G G A T C A C T G A C T G C T A G C T A G A G T C G A T C | KLF1(Zf)/HUDEP2-KLF1-CutnRun(GSE136251)/Homer | 1e-26 | -6.166e+01 | 0.0000 | 3638.0 | 23.10% | 6631.8 | 19.61% | motif file (matrix) | svg |
| 47 | A G T C A C G T A C T G A G C T A C G T A C G T G T C A A G T C | Foxo1(Forkhead)/RAW-Foxo1-ChIP-Seq(Fan\_et\_al.)/Homer | 1e-26 | -5.995e+01 | 0.0000 | 4310.0 | 27.37% | 8014.5 | 23.70% | motif file (matrix) | svg |
| 48 | T A C G T A G C G C T A C G A T C T A G A C G T C A G T C A G T G C T A A G T C G T C A G C A T | FOXK2(Forkhead)/U2OS-FOXK2-ChIP-Seq(E-MTAB-2204)/Homer | 1e-25 | -5.978e+01 | 0.0000 | 1399.0 | 8.88% | 2255.1 | 6.67% | motif file (matrix) | svg |
| 49 | G A C T C T A G A T G C A G T C G T C A T A C G A T G C A T C G | HIC1(Zf)/Treg-ZBTB29-ChIP-Seq(GSE99889)/Homer | 1e-25 | -5.886e+01 | 0.0000 | 5915.0 | 37.56% | 11347.8 | 33.55% | motif file (matrix) | svg |
| 50 | A G C T C A T G G C A T G A T C T G C A C T A G G A T C A C G T | Tgif2(Homeobox)/mES-Tgif2-ChIP-Seq(GSE55404)/Homer | 1e-25 | -5.821e+01 | 0.0000 | 7297.0 | 46.33% | 14268.4 | 42.19% | motif file (matrix) | svg |
| 51 | A G T C G A T C G C T A C G A T A C G T T A C G G C A T C T G A G A C T A C T G A G T C G C T A C T G A T C G A C A G T | Oct4:Sox17(POU,Homeobox,HMG)/F9-Sox17-ChIP-Seq(GSE44553)/Homer | 1e-25 | -5.797e+01 | 0.0000 | 372.0 | 2.36% | 442.5 | 1.31% | motif file (matrix) | svg |
| 52 | T C A G A G C T A T G C C G T A A G T C T C A G A C G T A T C G T C G A A G T C G A T C T G A C | TFE3(bHLH)/MEF-TFE3-ChIP-Seq(GSE75757)/Homer | 1e-24 | -5.693e+01 | 0.0000 | 343.0 | 2.18% | 400.0 | 1.18% | motif file (matrix) | svg |
| 53 | C T A G A C T G C T A G T C A G T C A G T A C G C T A G A C T G | Maz(Zf)/HepG2-Maz-ChIP-Seq(GSE31477)/Homer | 1e-24 | -5.687e+01 | 0.0000 | 4909.0 | 31.17% | 9279.4 | 27.44% | motif file (matrix) | svg |
| 54 | T A C G T C A G A G C T A T G C C G T A A G T C T C A G A C G T A C T G T C G A | USF1(bHLH)/GM12878-Usf1-ChIP-Seq(GSE32465)/Homer | 1e-24 | -5.533e+01 | 0.0000 | 1317.0 | 8.36% | 2128.7 | 6.29% | motif file (matrix) | svg |
| 55 | G A T C C T G A A G T C C G A T C G A T G A T C A G T C A C T G A T C G A G C T | Elk4(ETS)/Hela-Elk4-ChIP-Seq(GSE31477)/Homer | 1e-22 | -5.280e+01 | 0.0000 | 2001.0 | 12.71% | 3456.8 | 10.22% | motif file (matrix) | svg |
| 56 | A T G C A T G C A T C G T A C G A G C T A G T C G C T A A G T C T C A G G A C T A C T G T C G A | E-box(bHLH)/Promoter/Homer | 1e-22 | -5.203e+01 | 0.0000 | 347.0 | 2.20% | 418.0 | 1.24% | motif file (matrix) | svg |
| 57 | A G C T A G T C A G T C A C G T C T A G A C G T A C G T A C G T C G T A A G T C G A T C C G T A | FOXP1(Forkhead)/H9-FOXP1-ChIP-Seq(GSE31006)/Homer | 1e-22 | -5.091e+01 | 0.0000 | 981.0 | 6.23% | 1529.8 | 4.52% | motif file (matrix) | svg |
| 58 | T G C A C T G A A T G C G T C A A C G T A T G C A C G T A C T G A C T G T G C A | ZBTB18(Zf)/HEK293-ZBTB18.GFP-ChIP-Seq(GSE58341)/Homer | 1e-21 | -5.049e+01 | 0.0000 | 1456.0 | 9.25% | 2423.0 | 7.16% | motif file (matrix) | svg |
| 59 | C T A G T C A G C A G T T C A G A C T G A C T G G A T C C T A G A C T G C T A G T C A G A T G C | KLF14(Zf)/HEK293-KLF14.GFP-ChIP-Seq(GSE58341)/Homer | 1e-21 | -4.854e+01 | 0.0000 | 6010.0 | 38.16% | 11675.3 | 34.52% | motif file (matrix) | svg |
| 60 | T C A G G A C T C A G T C T G A A G C T C T A G G A C T T G C A C T G A A G T C | HLF(bZIP)/HSC-HLF.Flag-ChIP-Seq(GSE69817)/Homer | 1e-20 | -4.821e+01 | 0.0000 | 1560.0 | 9.91% | 2637.5 | 7.80% | motif file (matrix) | svg |
| 61 | T G A C C T G A C T A G C T G A C G T A A G T C C T G A A C G T G C A T T A G C G C A T A T C G G A C T G A C T G A T C | GRE(NR),IR3/RAW264.7-GRE-ChIP-Seq(Unpublished)/Homer | 1e-20 | -4.807e+01 | 0.0000 | 629.0 | 3.99% | 908.6 | 2.69% | motif file (matrix) | svg |
| 62 | T C G A C T A G A G T C A G T C C G T A C G T A A C G T T A G C T C A G T A C G | NFY(CCAAT)/Promoter/Homer | 1e-20 | -4.749e+01 | 0.0000 | 2340.0 | 14.86% | 4167.9 | 12.32% | motif file (matrix) | svg |
| 63 | A T G C C T G A A T C G T A C G A G T C C G A T T C A G C G A T C T A G A G C T G T C A G T C A C G T A A G T C C G T A T A C G C T G A | Fox:Ebox(Forkhead,bHLH)/Panc1-Foxa2-ChIP-Seq(GSE47459)/Homer | 1e-20 | -4.628e+01 | 0.0000 | 2286.0 | 14.52% | 4071.6 | 12.04% | motif file (matrix) | svg |
| 64 | C T A G A G T C T A C G T A C G T G A C C G T A A C T G T A G C G C A T C A T G A T G C A G C T | Ascl1(bHLH)/NeuralTubes-Ascl1-ChIP-Seq(GSE55840)/Homer | 1e-19 | -4.577e+01 | 0.0000 | 4440.0 | 28.19% | 8442.4 | 24.96% | motif file (matrix) | svg |
| 65 | C T A G G C A T G A T C C G T A A G T C T C A G G A C T C T A G | CLOCK(bHLH)/Liver-Clock-ChIP-Seq(GSE39860)/Homer | 1e-18 | -4.324e+01 | 0.0000 | 1491.0 | 9.47% | 2541.5 | 7.51% | motif file (matrix) | svg |
| 66 | A T G C G A C T A C G T C T A G A C G T A C G T A C G T C T G A G A T C G C T A A G C T C G T A | Foxa2(Forkhead)/Liver-Foxa2-ChIP-Seq(GSE25694)/Homer | 1e-18 | -4.283e+01 | 0.0000 | 1651.0 | 10.48% | 2854.8 | 8.44% | motif file (matrix) | svg |
| 67 | C T G A T G A C T G A C C G T A A C G T T G A C A G C T C T A G A C G T G A C T | Olig2(bHLH)/Neuron-Olig2-ChIP-Seq(GSE30882)/Homer | 1e-18 | -4.160e+01 | 0.0000 | 4804.0 | 30.50% | 9249.1 | 27.35% | motif file (matrix) | svg |
| 68 | C T G A T G C A T A G C T G A C T A C G T C A G C T G A G C T A T C A G G A C T | ELF1(ETS)/Jurkat-ELF1-ChIP-Seq(SRA014231)/Homer | 1e-17 | -4.010e+01 | 0.0000 | 1826.0 | 11.59% | 3219.9 | 9.52% | motif file (matrix) | svg |
| 69 | A C T G C A T G T C G A A C G T A C T G C G T A A T C G A C G T G T A C C G T A G A C T A G T C | Fos(bZIP)/TSC-Fos-ChIP-Seq(GSE110950)/Homer | 1e-17 | -3.948e+01 | 0.0000 | 1180.0 | 7.49% | 1972.3 | 5.83% | motif file (matrix) | svg |
| 70 | C T A G T A C G G A T C G T A C G C T A A G C T A G C T G T C A T C G A T A G C | Nanog(Homeobox)/mES-Nanog-ChIP-Seq(GSE11724)/Homer | 1e-16 | -3.894e+01 | 0.0000 | 8466.0 | 53.76% | 17038.0 | 50.38% | motif file (matrix) | svg |
| 71 | C A T G C T A G T C G A A C G T A C T G C G T A T A G C C G A T T G A C C G T A A G C T G A T C | Fra2(bZIP)/Striatum-Fra2-ChIP-Seq(GSE43429)/Homer | 1e-16 | -3.872e+01 | 0.0000 | 983.0 | 6.24% | 1604.4 | 4.74% | motif file (matrix) | svg |
| 72 | A G T C C T G A A G T C C G A T C A G T G A T C A T G C A C T G A T C G G A C T | Fli1(ETS)/CD8-FLI-ChIP-Seq(GSE20898)/Homer | 1e-16 | -3.736e+01 | 0.0000 | 3189.0 | 20.25% | 5980.6 | 17.68% | motif file (matrix) | svg |
| 73 | G C A T G C A T C T G A A C G T C T G A A C G T C G T A C G T A C G T A A G T C G T C A G T C A | Foxf1(Forkhead)/Lung-Foxf1-ChIP-Seq(GSE77951)/Homer | 1e-16 | -3.736e+01 | 0.0000 | 1814.0 | 11.52% | 3220.4 | 9.52% | motif file (matrix) | svg |
| 74 | G A T C T C G A A G T C C G A T C G A T A G T C A T G C A C T G A T C G G A C T | Elk1(ETS)/Hela-Elk1-ChIP-Seq(GSE31477)/Homer | 1e-16 | -3.731e+01 | 0.0000 | 1954.0 | 12.41% | 3497.5 | 10.34% | motif file (matrix) | svg |
| 75 | T G C A T A G C G A C T T G C A T G A C T G C A C G T A A G C T A G C T A G T C A G T C G T A C | GFY(?)/Promoter/Homer | 1e-16 | -3.713e+01 | 0.0000 | 272.0 | 1.73% | 339.0 | 1.00% | motif file (matrix) | svg |
| 76 | T C G A A C G T C A T G G C T A T A G C C G A T G T A C G C T A A C G T A T G C | AP-1(bZIP)/ThioMac-PU.1-ChIP-Seq(GSE21512)/Homer | 1e-16 | -3.699e+01 | 0.0000 | 1479.0 | 9.39% | 2567.4 | 7.59% | motif file (matrix) | svg |
| 77 | T C A G C T A G A T G C G A C T T G C A C G T A A G C T A C G T C T G A T A C G | En1(Homeobox)/SUM149-EN1-ChIP-Seq(GSE120957)/Homer | 1e-15 | -3.644e+01 | 0.0000 | 3667.0 | 23.28% | 6969.2 | 20.61% | motif file (matrix) | svg |
| 78 | G A C T A G T C C G A T A C T G C T G A T G A C G T A C C G T A A T C G G C A T C T G A C T A G | Bcl11a(Zf)/HSPC-BCL11A-ChIP-Seq(GSE104676)/Homer | 1e-15 | -3.567e+01 | 0.0000 | 1728.0 | 10.97% | 3066.1 | 9.07% | motif file (matrix) | svg |
| 79 | T G C A T C G A T A G C G T A C T C A G C T A G G T C A G C T A T C A G G A C T | ETS(ETS)/Promoter/Homer | 1e-15 | -3.565e+01 | 0.0000 | 1214.0 | 7.71% | 2065.6 | 6.11% | motif file (matrix) | svg |
| 80 | G C T A T C G A C G T A C T A G A G C T G T C A G T C A C G T A A G T C C G T A | FOXA1(Forkhead)/LNCAP-FOXA1-ChIP-Seq(GSE27824)/Homer | 1e-15 | -3.492e+01 | 0.0000 | 2451.0 | 15.56% | 4514.9 | 13.35% | motif file (matrix) | svg |
| 81 | T A C G A T G C G A C T A C T G A G C T A G T C G T C A T G C A A C G T A G T C G C T A T G C A | Pknox1(Homeobox)/ES-Prep1-ChIP-Seq(GSE63282)/Homer | 1e-14 | -3.425e+01 | 0.0000 | 679.0 | 4.31% | 1065.7 | 3.15% | motif file (matrix) | svg |
| 82 | T A G C T A G C G A C T C T A G A G C T A G T C G T C A T G C A A C G T A T G C G C T A T G C A | Pbx3(Homeobox)/GM12878-PBX3-ChIP-Seq(GSE32465)/Homer | 1e-14 | -3.415e+01 | 0.0000 | 708.0 | 4.50% | 1119.1 | 3.31% | motif file (matrix) | svg |
| 83 | C T G A A C T G A C T G A G T C A G T C A G C T C T A G T A C G | ZFX(Zf)/mES-Zfx-ChIP-Seq(GSE11431)/Homer | 1e-14 | -3.372e+01 | 0.0000 | 3614.0 | 22.95% | 6896.1 | 20.39% | motif file (matrix) | svg |
| 84 | A C T G C T A G T C G A C G A T C A T G G C T A A T C G C G A T G T A C G C T A A G C T G T A C | Fra1(bZIP)/BT549-Fra1-ChIP-Seq(GSE46166)/Homer | 1e-14 | -3.359e+01 | 0.0000 | 1083.0 | 6.88% | 1829.4 | 5.41% | motif file (matrix) | svg |
| 85 | C T A G T C G A G C A T C A T G G C T A T A G C C G A T G T A C C T G A A G C T | JunB(bZIP)/DendriticCells-Junb-ChIP-Seq(GSE36099)/Homer | 1e-14 | -3.350e+01 | 0.0000 | 1103.0 | 7.00% | 1868.7 | 5.53% | motif file (matrix) | svg |
| 86 | T G C A A G C T C A T G C G T A A G C T A C T G G A T C G T C A C G T A A G C T | Atf4(bZIP)/MEF-Atf4-ChIP-Seq(GSE35681)/Homer | 1e-14 | -3.330e+01 | 0.0000 | 529.0 | 3.36% | 797.1 | 2.36% | motif file (matrix) | svg |
| 87 | C G T A C T G A C T A G C G T A C G T A A G T C C G T A C A G T G C A T G T C A C G A T A C T G A C G T G C A T G A T C | PGR(NR)/EndoStromal-PGR-ChIP-Seq(GSE69539)/Homer | 1e-14 | -3.293e+01 | 0.0000 | 564.0 | 3.58% | 862.4 | 2.55% | motif file (matrix) | svg |
| 88 | A T G C T A C G A G T C G A C T T G C A C T G A G A C T A C G T C T G A T C A G | LHX9(Homeobox)/Hct116-LHX9.V5-ChIP-Seq(GSE116822)/Homer | 1e-13 | -3.213e+01 | 0.0000 | 2755.0 | 17.49% | 5161.5 | 15.26% | motif file (matrix) | svg |
| 89 | C G A T T A C G G A T C G C T A T C A G G A C T C G A T G T A C G A T C G T C A T C G A T G A C C G T A C T A G G A C T C T A G C T A G G T A C A G T C C G T A | CTCF-SatelliteElement(Zf?)/CD4+-CTCF-ChIP-Seq(Barski\_et\_al.)/Homer | 1e-13 | -3.210e+01 | 0.0000 | 96.0 | 0.61% | 84.2 | 0.25% | motif file (matrix) | svg |
| 90 | A G C T G C A T A C T G A C G T A G T C A C G T C T A G T A C G | Smad3(MAD)/NPC-Smad3-ChIP-Seq(GSE36673)/Homer | 1e-13 | -3.194e+01 | 0.0000 | 7149.0 | 45.39% | 14334.0 | 42.38% | motif file (matrix) | svg |
| 91 | T A C G C T G A C T A G T C G A C G T A A G T C C T G A A T C G G C A T T A G C G A C T A C T G A C G T A G C T A G T C G A C T | GRE(NR),IR3/A549-GR-ChIP-Seq(GSE32465)/Homer | 1e-13 | -3.189e+01 | 0.0000 | 381.0 | 2.42% | 541.7 | 1.60% | motif file (matrix) | svg |
| 92 | G A C T G C A T C T A G C G A T G A T C T C G A C A T G G A T C | Tgif1(Homeobox)/mES-Tgif1-ChIP-Seq(GSE55404)/Homer | 1e-13 | -3.184e+01 | 0.0000 | 6700.0 | 42.54% | 13381.3 | 39.57% | motif file (matrix) | svg |
| 93 | C T A G T C G A A C G T A C T G C G T A A T G C A C G T G T A C C G T A A G C T G A T C G T A C | Atf3(bZIP)/GBM-ATF3-ChIP-Seq(GSE33912)/Homer | 1e-13 | -3.179e+01 | 0.0000 | 1286.0 | 8.17% | 2235.1 | 6.61% | motif file (matrix) | svg |
| 94 | T C G A T C G A T A G C G T A C T C A G T A C G C G T A C G T A T C A G A G C T | GABPA(ETS)/Jurkat-GABPa-ChIP-Seq(GSE17954)/Homer | 1e-13 | -3.074e+01 | 0.0000 | 2580.0 | 16.38% | 4823.3 | 14.26% | motif file (matrix) | svg |
| 95 | T C G A C G T A A G T C A G C T C G T A A G T C T C G A G C T A G A C T C G A T A G T C A G T C A G T C C T G A T C A G T G C A T C G A C A G T A T C G A G T C | GFY-Staf(?,Zf)/Promoter/Homer | 1e-12 | -2.990e+01 | 0.0000 | 250.0 | 1.59% | 325.6 | 0.96% | motif file (matrix) | svg |
| 96 | T C G A G C A T A C G T C T A G G T A C T C G A G C A T T G A C T C G A A C G T | Chop(bZIP)/MEF-Chop-ChIP-Seq(GSE35681)/Homer | 1e-12 | -2.876e+01 | 0.0000 | 408.0 | 2.59% | 602.7 | 1.78% | motif file (matrix) | svg |
| 97 | T A G C G T A C C G T A C T A G A C T G T G C A C G T A A T G C C G T A A T C G | AR-halfsite(NR)/LNCaP-AR-ChIP-Seq(GSE27824)/Homer | 1e-12 | -2.837e+01 | 0.0000 | 8824.0 | 56.03% | 17989.8 | 53.19% | motif file (matrix) | svg |
| 98 | A G T C G C A T C G T A C G T A G T A C A C G T A C T G G A T C G A T C T C G A | BMYB(HTH)/Hela-BMYB-ChIP-Seq(GSE27030)/Homer | 1e-12 | -2.823e+01 | 0.0000 | 3396.0 | 21.56% | 6524.6 | 19.29% | motif file (matrix) | svg |
| 99 | A T G C A G C T T C A G T G A C T C A G A T G C T G C A A C G T A T C G G A T C A C T G A G T C | NRF1(NRF)/MCF7-NRF1-ChIP-Seq(Unpublished)/Homer | 1e-11 | -2.746e+01 | 0.0000 | 827.0 | 5.25% | 1385.7 | 4.10% | motif file (matrix) | svg |
| 100 | T A G C G T A C A G T C G T A C C G A T A G T C A G T C A G T C A G T C A G T C C G T A G A T C | Zfp281(Zf)/ES-Zfp281-ChIP-Seq(GSE81042)/Homer | 1e-11 | -2.705e+01 | 0.0000 | 1012.0 | 6.43% | 1743.9 | 5.16% | motif file (matrix) | svg |
| 101 | C T A G T C G A C G A T A C T G C G T A T A C G A G C T T G A C G C T A A C G T G A T C T A G C | Fosl2(bZIP)/3T3L1-Fosl2-ChIP-Seq(GSE56872)/Homer | 1e-11 | -2.704e+01 | 0.0000 | 705.0 | 4.48% | 1157.4 | 3.42% | motif file (matrix) | svg |
| 102 | C A G T T C A G A G C T G A C T A C G T A G T C G A T C G A C T C T G A A C T G G A T C C G T A C T G A A G T C G T A C | Rfx6(HTH)/Min6b1-Rfx6.HA-ChIP-Seq(GSE62844)/Homer | 1e-11 | -2.625e+01 | 0.0000 | 2884.0 | 18.31% | 5501.5 | 16.27% | motif file (matrix) | svg |
| 103 | C G T A C T A G A C T G A C T G G A C T C T A G C A G T C T A G C A T G G A T C | KLF5(Zf)/LoVo-KLF5-ChIP-Seq(GSE49402)/Homer | 1e-11 | -2.620e+01 | 0.0000 | 4529.0 | 28.76% | 8906.1 | 26.33% | motif file (matrix) | svg |
| 104 | C A G T T G C A A C G T A C T G C G T A A T C G C G A T T G A C C G T A A C G T | BATF(bZIP)/Th17-BATF-ChIP-Seq(GSE39756)/Homer | 1e-11 | -2.607e+01 | 0.0000 | 1280.0 | 8.13% | 2274.3 | 6.73% | motif file (matrix) | svg |
| 105 | C A T G G T A C C G T A A G T C C T A G A C G T A C T G G T A C A G T C A G C T | bHLHE40(bHLH)/HepG2-BHLHE40-ChIP-Seq(GSE31477)/Homer | 1e-11 | -2.577e+01 | 0.0000 | 854.0 | 5.42% | 1449.8 | 4.29% | motif file (matrix) | svg |
| 106 | C G T A G C T A C G A T C T A G A C G T G T C A C G T A C G T A A G T C C G T A T G C A T A C G | FoxL2(Forkhead)/Ovary-FoxL2-ChIP-Seq(GSE60858)/Homer | 1e-11 | -2.576e+01 | 0.0000 | 1627.0 | 10.33% | 2964.6 | 8.77% | motif file (matrix) | svg |
| 107 | C T A G T C G A A C G T A C T G C G T A T A G C C G A T G T A C C G T A A G C T G A T C G T A C | Jun-AP1(bZIP)/K562-cJun-ChIP-Seq(GSE31477)/Homer | 1e-10 | -2.512e+01 | 0.0000 | 519.0 | 3.30% | 822.2 | 2.43% | motif file (matrix) | svg |
| 108 | T C G A C A G T A C T G A G C T C G T A C G T A A C G T A C G T C T G A T A G C | Dlx3(Homeobox)/Kerainocytes-Dlx3-ChIP-Seq(GSE89884)/Homer | 1e-10 | -2.467e+01 | 0.0000 | 1150.0 | 7.30% | 2032.6 | 6.01% | motif file (matrix) | svg |
| 109 | T G C A A C T G T A C G A T G C A G T C G A C T T C G A A T C G | ZNF711(Zf)/SHSY5Y-ZNF711-ChIP-Seq(GSE20673)/Homer | 1e-10 | -2.455e+01 | 0.0000 | 5275.0 | 33.49% | 10499.6 | 31.05% | motif file (matrix) | svg |
| 110 | A T C G A G C T A C T G A G T C A C T G A G T C C G T A A C G T A C T G A G T C A C T G A G T C | NRF(NRF)/Promoter/Homer | 1e-10 | -2.437e+01 | 0.0000 | 878.0 | 5.57% | 1506.5 | 4.45% | motif file (matrix) | svg |
| 111 | T A G C C G T A C T G A T A C G C G T A A C G T A C T G A C T G A G T C T A C G C T A G G T A C | YY1(Zf)/Promoter/Homer | 1e-10 | -2.369e+01 | 0.0000 | 375.0 | 2.38% | 567.9 | 1.68% | motif file (matrix) | svg |
| 112 | A T G C T A C G A G C T T G C A C G T A C G A T A C G T C T G A | DLX5(Homeobox)/BasalGanglia-Dlx5-ChIP-seq(GSE124936)/Homer | 1e-10 | -2.365e+01 | 0.0000 | 1396.0 | 8.86% | 2527.9 | 7.47% | motif file (matrix) | svg |
| 113 | A T G C A G T C A G C T A G C T A C G T A T C G C G T A C G A T T A G C G A C T | LEF1(HMG)/H1-LEF1-ChIP-Seq(GSE64758)/Homer | 1e-10 | -2.365e+01 | 0.0000 | 1480.0 | 9.40% | 2694.2 | 7.97% | motif file (matrix) | svg |
| 114 | C T A G C T A G A G T C T C A G A C T G A C G T A C G T C T G A | MYB(HTH)/ERMYB-Myb-ChIPSeq(GSE22095)/Homer | 1e-10 | -2.341e+01 | 0.0000 | 4115.0 | 26.13% | 8090.5 | 23.92% | motif file (matrix) | svg |
| 115 | C T G A C T A G T C G A C G T A A T G C C G T A A T C G C G A T T A G C G C A T A T C G G C A T A G C T G A T C G A C T A G C T | ARE(NR)/LNCAP-AR-ChIP-Seq(GSE27824)/Homer | 1e-9 | -2.218e+01 | 0.0000 | 740.0 | 4.70% | 1258.2 | 3.72% | motif file (matrix) | svg |
| 116 | C G A T T A C G T G A C G A C T C A T G C G T A T A C G A C G T G T A C C T G A | Bach2(bZIP)/OCILy7-Bach2-ChIP-Seq(GSE44420)/Homer | 1e-9 | -2.180e+01 | 0.0000 | 456.0 | 2.90% | 725.4 | 2.14% | motif file (matrix) | svg |
| 117 | G C T A A G C T T A C G G T C A C T G A C G A T C T G A G C A T C A G T A G T C | Brn2(POU,Homeobox)/NPC-Brn2-ChIP-Seq(GSE35496)/Homer | 1e-9 | -2.156e+01 | 0.0000 | 182.0 | 1.16% | 239.8 | 0.71% | motif file (matrix) | svg |
| 118 | C T A G A T G C A T G C C G A T A C T G G A C T A T G C G C T A T G A C A G C T T A G C G C T A | PBX1(Homeobox)/MCF7-PBX1-ChIP-Seq(GSE28007)/Homer | 1e-9 | -2.151e+01 | 0.0000 | 275.0 | 1.75% | 399.4 | 1.18% | motif file (matrix) | svg |
| 119 | C G T A C G T A C G T A G C A T G C A T T A C G G T A C G A C T A C T G C G T A A T C G A C G T G T A C C G T A A G C T | Bach1(bZIP)/K562-Bach1-ChIP-Seq(GSE31477)/Homer | 1e-9 | -2.124e+01 | 0.0000 | 138.0 | 0.88% | 168.6 | 0.50% | motif file (matrix) | svg |
| 120 | C A T G A C T G A G C T A T G C C G T A A T G C G T A C G A C T T A C G C T G A A C T G A C T G G C A T A T G C C T G A | THRb(NR)/HepG2-THRb.Flag-ChIP-Seq(Encode)/Homer | 1e-9 | -2.097e+01 | 0.0000 | 1540.0 | 9.78% | 2844.7 | 8.41% | motif file (matrix) | svg |
| 121 | G C T A T C G A C G T A C T A G A G C T G T C A G T C A C G T A A G T C C G T A | FOXA1(Forkhead)/MCF7-FOXA1-ChIP-Seq(GSE26831)/Homer | 1e-9 | -2.079e+01 | 0.0000 | 1957.0 | 12.43% | 3687.2 | 10.90% | motif file (matrix) | svg |
| 122 | C T G A T A G C T G A C T C A G C T A G G T C A C G T A T C A G A G C T T C A G | ETV4(ETS)/HepG2-ETV4-ChIP-Seq(ENCODE)/Homer | 1e-8 | -2.038e+01 | 0.0000 | 3261.0 | 20.71% | 6368.2 | 18.83% | motif file (matrix) | svg |
| 123 | G A C T C T A G C T A G A G T C T G C A A C T G A C G T A C G T C T A G T C A G | AMYB(HTH)/Testes-AMYB-ChIP-Seq(GSE44588)/Homer | 1e-8 | -1.975e+01 | 0.0000 | 3528.0 | 22.40% | 6933.9 | 20.50% | motif file (matrix) | svg |
| 124 | A G C T G T C A C G T A A C G T A C G T C T G A T C A G A T G C | Lhx2(Homeobox)/HFSC-Lhx2-ChIP-Seq(GSE48068)/Homer | 1e-8 | -1.953e+01 | 0.0000 | 1953.0 | 12.40% | 3696.6 | 10.93% | motif file (matrix) | svg |
| 125 | C T A G A G C T G A C T C A T G A G T C A G T C G T C A C A G T C T A G T C A G G T A C C T G A T C G A G A T C T G A C | Rfx2(HTH)/LoVo-RFX2-ChIP-Seq(GSE49402)/Homer | 1e-8 | -1.929e+01 | 0.0000 | 319.0 | 2.03% | 488.9 | 1.45% | motif file (matrix) | svg |
| 126 | C T A G T A C G G A T C G T C A T G C A A C G T T G C A G C T A T C G A T G C A | Hoxa9(Homeobox)/ChickenMSG-Hoxa9.Flag-ChIP-Seq(GSE86088)/Homer | 1e-8 | -1.909e+01 | 0.0000 | 5337.0 | 33.89% | 10737.6 | 31.75% | motif file (matrix) | svg |
| 127 | G T A C C A G T A C T G A C T G A C T G G A T C A C T G A C G T A C T G A C T G A G T C G A T C | KLF6(Zf)/PDAC-KLF6-ChIP-Seq(GSE64557)/Homer | 1e-8 | -1.877e+01 | 0.0000 | 3738.0 | 23.73% | 7388.5 | 21.85% | motif file (matrix) | svg |
| 128 | C A T G G T A C G A C T G C T A C G T A C G T A C G T A G C T A G A C T C T G A T C A G G T A C | Mef2c(MADS)/GM12878-Mef2c-ChIP-Seq(GSE32465)/Homer | 1e-7 | -1.817e+01 | 0.0000 | 754.0 | 4.79% | 1318.8 | 3.90% | motif file (matrix) | svg |
| 129 | A G T C T A G C G A C T A C G T C T A G A C G T A C G T A C G T C T G A A G T C G C T A G A C T C G T A C T A G A C T G | Foxa3(Forkhead)/Liver-Foxa3-ChIP-Seq(GSE77670)/Homer | 1e-7 | -1.800e+01 | 0.0000 | 626.0 | 3.97% | 1072.3 | 3.17% | motif file (matrix) | svg |
| 130 | T C A G T G A C G T A C T G C A G T A C C T A G G T A C A T G C A G T C G T C A A G T C G A C T | Klf9(Zf)/GBM-Klf9-ChIP-Seq(GSE62211)/Homer | 1e-7 | -1.761e+01 | 0.0000 | 1694.0 | 10.76% | 3197.5 | 9.45% | motif file (matrix) | svg |
| 131 | C T G A A G T C C G A T A G C T A T G C G T A C A C G T A T C G C A G T G C A T | Elf4(ETS)/BMDM-Elf4-ChIP-Seq(GSE88699)/Homer | 1e-7 | -1.748e+01 | 0.0000 | 2684.0 | 17.04% | 5225.4 | 15.45% | motif file (matrix) | svg |
| 132 | A T C G A T C G A T G C G A C T T C G A C G T A G C A T A G C T C T G A T A C G | DLX2(Homeobox)/BasalGanglia-Dlx2-ChIP-seq(GSE124936)/Homer | 1e-7 | -1.716e+01 | 0.0000 | 2768.0 | 17.58% | 5404.9 | 15.98% | motif file (matrix) | svg |
| 133 | C G A T C T A G A C G T A C G T A C G T C G T A A G C T C G A T A G C T C G T A C T A G T A G C | FoxD3(forkhead)/ZebrafishEmbryo-Foxd3.biotin-ChIP-seq(GSE106676)/Homer | 1e-7 | -1.696e+01 | 0.0000 | 1820.0 | 11.56% | 3462.4 | 10.24% | motif file (matrix) | svg |
| 134 | T A C G T C G A C A G T A C T G G C T A A T G C C G A T G T A C C G T A A C T G T A G C C G T A | NF-E2(bZIP)/K562-NFE2-ChIP-Seq(GSE31477)/Homer | 1e-7 | -1.689e+01 | 0.0000 | 144.0 | 0.91% | 191.4 | 0.57% | motif file (matrix) | svg |
| 135 | A T C G A T G C A G T C T A G C G A C T T C G A G C T A G C A T A G C T C T G A | DLX1(Homeobox)/BasalGanglia-Dlx1-ChIP-seq(GSE124936)/Homer | 1e-7 | -1.683e+01 | 0.0000 | 2471.0 | 15.69% | 4797.7 | 14.19% | motif file (matrix) | svg |
| 136 | T G A C G C T A T G A C C G T A T C A G G A T C C G T A C A T G C A T G C T A G C T A G C T A G | Unknown-ESC-element(?)/mES-Nanog-ChIP-Seq(GSE11724)/Homer | 1e-7 | -1.659e+01 | 0.0000 | 1612.0 | 10.24% | 3045.3 | 9.00% | motif file (matrix) | svg |
| 137 | G T C A A C G T C T A G G T C A C G T A G C A T C G T A C G T A G C A T C A G T A G T C C G T A C A G T C T A G C T G A | OCT:OCT(POU,Homeobox,IR1)/NPC-Brn2-ChIP-Seq(GSE35496)/Homer | 1e-7 | -1.657e+01 | 0.0000 | 46.0 | 0.29% | 40.7 | 0.12% | motif file (matrix) | svg |
| 138 | T C A G T A C G T A G C A C G T A C T G C G A T A G T C C G T A T A C G A G T C | Meis1(Homeobox)/MastCells-Meis1-ChIP-Seq(GSE48085)/Homer | 1e-7 | -1.655e+01 | 0.0000 | 4142.0 | 26.30% | 8277.0 | 24.47% | motif file (matrix) | svg |
| 139 | T C G A T A G C G T C A A C T G A C T G C G T A C G T A C T A G A G C T T C A G | ERG(ETS)/VCaP-ERG-ChIP-Seq(GSE14097)/Homer | 1e-7 | -1.644e+01 | 0.0000 | 3917.0 | 24.87% | 7808.7 | 23.09% | motif file (matrix) | svg |
| 140 | C A T G C T A G A G C T G A C T C A T G A G T C G A T C G C T A C G A T C T A G T C A G G T A C C T G A T C G A | X-box(HTH)/NPC-H3K4me1-ChIP-Seq(GSE16256)/Homer | 1e-7 | -1.632e+01 | 0.0000 | 313.0 | 1.99% | 493.3 | 1.46% | motif file (matrix) | svg |
| 141 | T C G A T A G C T G C A A C T G A C T G C G T A C G T A C T A G G A C T T A C G | ETS1(ETS)/Jurkat-ETS1-ChIP-Seq(GSE17954)/Homer | 1e-7 | -1.632e+01 | 0.0000 | 2754.0 | 17.49% | 5390.7 | 15.94% | motif file (matrix) | svg |
| 142 | A G T C A T C G C T A G A G C T G A C T C T A G A G T C A G T C G C T A C A G T T C A G T C A G G A T C C T G A T C G A G A T C | RFX(HTH)/K562-RFX3-ChIP-Seq(SRA012198)/Homer | 1e-7 | -1.630e+01 | 0.0000 | 290.0 | 1.84% | 451.4 | 1.33% | motif file (matrix) | svg |
| 143 | G T A C G A C T C G T A C T G A T C G A C G T A G C T A C A G T C T G A T A C G | Mef2a(MADS)/HL1-Mef2a.biotin-ChIP-Seq(GSE21529)/Homer | 1e-6 | -1.595e+01 | 0.0000 | 736.0 | 4.67% | 1303.2 | 3.85% | motif file (matrix) | svg |
| 144 | T G C A A G C T A C G T C T A G G A T C C T A G G A T C G T C A C T G A A G T C | CEBP(bZIP)/ThioMac-CEBPb-ChIP-Seq(GSE21512)/Homer | 1e-6 | -1.593e+01 | 0.0000 | 1168.0 | 7.42% | 2159.7 | 6.39% | motif file (matrix) | svg |
| 145 | C T A G T C G A C G A T C T A G G C A T C A G T C T A G G A T C C G T A G T C A | CEBP:AP1(bZIP)/ThioMac-CEBPb-ChIP-Seq(GSE21512)/Homer | 1e-6 | -1.579e+01 | 0.0000 | 1398.0 | 8.88% | 2623.7 | 7.76% | motif file (matrix) | svg |
| 146 | C A T G A G T C G A C T C G T A C G A T G C A T G A C T G C A T C G A T C T A G C A T G T G A C | Mef2b(MADS)/HEK293-Mef2b.V5-ChIP-Seq(GSE67450)/Homer | 1e-6 | -1.574e+01 | 0.0000 | 1462.0 | 9.28% | 2754.0 | 8.14% | motif file (matrix) | svg |
| 147 | T C G A G A C T A G C T T G A C A G C T G T A C T C A G G A T C A T C G T G C A A C T G C T G A | GFX(?)/Promoter/Homer | 1e-6 | -1.531e+01 | 0.0000 | 84.0 | 0.53% | 98.6 | 0.29% | motif file (matrix) | svg |
| 148 | A C T G T C A G A G C T G A C T C A T G A G T C A G T C G C T A C G A T C T A G T C A G G T A C C T G A T C G A | Rfx1(HTH)/NPC-H3K4me1-ChIP-Seq(GSE16256)/Homer | 1e-6 | -1.488e+01 | 0.0000 | 550.0 | 3.49% | 951.5 | 2.81% | motif file (matrix) | svg |
| 149 | G A C T A G C T G T A C G A C T C T G A A C T G G T C A C T G A A T G C T A C G G A C T A C G T A G T C G A C T C T G A | HRE(HSF)/Striatum-HSF1-ChIP-Seq(GSE38000)/Homer | 1e-6 | -1.469e+01 | 0.0000 | 457.0 | 2.90% | 774.5 | 2.29% | motif file (matrix) | svg |
| 150 | C G T A C G T A A G C T G A C T T G C A G T C A A C G T A G C T C T G A T C A G | Lhx3(Homeobox)/Neuron-Lhx3-ChIP-Seq(GSE31456)/Homer | 1e-6 | -1.461e+01 | 0.0000 | 3020.0 | 19.18% | 5974.9 | 17.67% | motif file (matrix) | svg |
| 151 | C A T G G A C T G C A T A C T G A G C T A C T G A C T G C G T A G C A T A G C T A T C G T A C G | Foxh1(Forkhead)/hESC-FOXH1-ChIP-Seq(GSE29422)/Homer | 1e-6 | -1.450e+01 | 0.0000 | 1200.0 | 7.62% | 2241.5 | 6.63% | motif file (matrix) | svg |
| 152 | C A T G A G T C A G C T C G T A C G A T C G A T G C A T G C A T C G A T C T G A C A T G T G A C | Mef2d(MADS)/Retina-Mef2d-ChIP-Seq(GSE61391)/Homer | 1e-6 | -1.445e+01 | 0.0000 | 335.0 | 2.13% | 545.0 | 1.61% | motif file (matrix) | svg |
| 153 | T C A G A T C G G A C T A C T G G A C T C A G T C T A G C G T A G T A C C G T A C T A G A T C G | Tbx20(T-box)/Heart-Tbx20-ChIP-Seq(GSE29636)/Homer | 1e-6 | -1.433e+01 | 0.0000 | 577.0 | 3.66% | 1008.3 | 2.98% | motif file (matrix) | svg |
| 154 | T A C G T A C G G T A C A T C G T A C G T A C G G T C A C T G A C G T A G A C T | E2F4(E2F)/K562-E2F4-ChIP-Seq(GSE31477)/Homer | 1e-6 | -1.417e+01 | 0.0000 | 1555.0 | 9.87% | 2964.3 | 8.77% | motif file (matrix) | svg |
| 155 | C T A G T A C G G A T C G T C A T C G A A C G T T C A G C G T A C G T A C G T A | Hoxd10(Homeobox)/ChickenMSG-Hoxd10.Flag-ChIP-Seq(GSE86088)/Homer | 1e-6 | -1.397e+01 | 0.0000 | 2226.0 | 14.13% | 4342.1 | 12.84% | motif file (matrix) | svg |
| 156 | C G T A C A T G A G T C G A C T T G C A C G T A A C G T A C G T C T G A T C A G | Lhx1(Homeobox)/EmbryoCarcinoma-Lhx1-ChIP-Seq(GSE70957)/Homer | 1e-5 | -1.341e+01 | 0.0000 | 1971.0 | 12.52% | 3827.9 | 11.32% | motif file (matrix) | svg |
| 157 | T C G A T A G C T G A C C T G A A G T C A C T G G A C T C A T G | c-Myc(bHLH)/LNCAP-cMyc-ChIP-Seq(Unpublished)/Homer | 1e-5 | -1.337e+01 | 0.0000 | 1337.0 | 8.49% | 2533.3 | 7.49% | motif file (matrix) | svg |
| 158 | T C G A G C A T A T G C C T G A A T G C T A G C A G T C G T A C T C G A A G C T | Srebp1a(bHLH)/HepG2-Srebp1a-ChIP-Seq(GSE31477)/Homer | 1e-5 | -1.300e+01 | 0.0000 | 460.0 | 2.92% | 793.7 | 2.35% | motif file (matrix) | svg |
| 159 | T G C A C T G A A G T C G T C A A C T G A C T G C G T A C G T A C T G A A G C T | EWS:FLI1-fusion(ETS)/SK\_N\_MC-EWS:FLI1-ChIP-Seq(SRA014231)/Homer | 1e-5 | -1.286e+01 | 0.0000 | 1529.0 | 9.71% | 2931.2 | 8.67% | motif file (matrix) | svg |
| 160 | C T A G T A C G G A C T T G C A T G C A C G A T T A C G C T G A T C G A C T G A | Hoxa10(Homeobox)/ChickenMSG-Hoxa10.Flag-ChIP-Seq(GSE86088)/Homer | 1e-5 | -1.272e+01 | 0.0000 | 1097.0 | 6.97% | 2057.2 | 6.08% | motif file (matrix) | svg |
| 161 | A C G T C T A G A G C T A C G T A C G T C T G A A G T C G A C T A G C T C G T A | FOXM1(Forkhead)/MCF7-FOXM1-ChIP-Seq(GSE72977)/Homer | 1e-5 | -1.251e+01 | 0.0000 | 2073.0 | 13.16% | 4054.0 | 11.99% | motif file (matrix) | svg |
| 162 | A G T C G A C T C A G T G T A C A G T C A T C G T C A G A C T G G T C A C G T A | Stat3(Stat)/mES-Stat3-ChIP-Seq(GSE11431)/Homer | 1e-5 | -1.250e+01 | 0.0000 | 1240.0 | 7.87% | 2349.6 | 6.95% | motif file (matrix) | svg |
| 163 | T G A C C T G A C T A G T C G A C T G A A T G C C G T A A C T G G C A T G T A C G C A T A T C G G C A T A G C T G A T C | PR(NR)/T47D-PR-ChIP-Seq(GSE31130)/Homer | 1e-5 | -1.237e+01 | 0.0000 | 5062.0 | 32.14% | 10314.7 | 30.50% | motif file (matrix) | svg |
| 164 | T C G A C T G A T A G C T G A C T C A G T C A G C G T A C G T A T C A G A G C T | ETV1(ETS)/GIST48-ETV1-ChIP-Seq(GSE22441)/Homer | 1e-5 | -1.236e+01 | 0.0000 | 3580.0 | 22.73% | 7192.3 | 21.27% | motif file (matrix) | svg |
| 165 | T A C G C T G A C A T G G A T C G T A C G C A T T C A G T A C G A G C T G T C A G A T C G C A T T A C G C G T A C T A G G A T C G A T C C G A T A C T G T C A G | ZNF322(Zf)/HEK293-ZNF322.GFP-ChIP-Seq(GSE58341)/Homer | 1e-5 | -1.230e+01 | 0.0000 | 828.0 | 5.26% | 1523.4 | 4.50% | motif file (matrix) | svg |
| 166 | C T A G G T A C A G T C T G C A A G T C C T G A A G T C A G T C A G T C G C T A | Klf4(Zf)/mES-Klf4-ChIP-Seq(GSE11431)/Homer | 1e-5 | -1.213e+01 | 0.0000 | 1327.0 | 8.43% | 2531.5 | 7.49% | motif file (matrix) | svg |
| 167 | T G C A C T A G C T A G C T G A C A T G A C T G T G C A G A T C G T C A T G C A G T C A G T C A A G C T C T A G G C A T | ZNF675(Zf)/HEK293-ZNF675.GFP-ChIP-Seq(GSE58341)/Homer | 1e-5 | -1.209e+01 | 0.0000 | 316.0 | 2.01% | 525.7 | 1.55% | motif file (matrix) | svg |
| 168 | G C T A C G A T C T A G G T A C G C T A C G A T C G T A G C T A C G A T C A G T G A T C G C T A | Pit1+1bp(Homeobox)/GCrat-Pit1-ChIP-Seq(GSE58009)/Homer | 1e-5 | -1.196e+01 | 0.0000 | 551.0 | 3.50% | 979.9 | 2.90% | motif file (matrix) | svg |
| 169 | G A C T C A G T A G C T C G A T A G T C G A T C A G T C C G T A A T G C T C A G | Rbpj1(?)/Panc1-Rbpj1-ChIP-Seq(GSE47459)/Homer | 1e-5 | -1.183e+01 | 0.0000 | 3343.0 | 21.23% | 6708.7 | 19.84% | motif file (matrix) | svg |
| 170 | A C T G G T C A A C G T G C A T C G A T T C A G G T A C G T C A A C G T C T G A | Oct11(POU,Homeobox)/NCIH1048-POU2F3-ChIP-seq(GSE115123)/Homer | 1e-5 | -1.175e+01 | 0.0000 | 607.0 | 3.85% | 1091.8 | 3.23% | motif file (matrix) | svg |
| 171 | A C T G A C G T C A T G A T C G A T C G T G A C A C T G A T C G A T C G T G C A C T G A C G T A | E2F3(E2F)/MEF-E2F3-ChIP-Seq(GSE71376)/Homer | 1e-5 | -1.173e+01 | 0.0000 | 2093.0 | 13.29% | 4109.9 | 12.15% | motif file (matrix) | svg |
| 172 | G T C A G C A T A C T G G T A C G A C T A C T G G C T A A T C G C A G T G T A C C G T A A G C T | Nrf2(bZIP)/Lymphoblast-Nrf2-ChIP-Seq(GSE37589)/Homer | 1e-5 | -1.153e+01 | 0.0000 | 122.0 | 0.77% | 173.4 | 0.51% | motif file (matrix) | svg |
| 173 | A T G C T A G C A G C T A G C T T G A C G A C T T C A G T A C G G T C A C T G A A T C G T A G C G A C T C A G T A G T C A G C T T C G A A T C G T G C A T G C A | HRE(HSF)/HepG2-HSF1-ChIP-Seq(GSE31477)/Homer | 1e-4 | -1.146e+01 | 0.0000 | 375.0 | 2.38% | 642.1 | 1.90% | motif file (matrix) | svg |
| 174 | T C A G G C T A T C A G C A G T T G A C G T C A A G T C A T C G T G C A G T A C C A G T G A T C | Npas4(bHLH)/Neuron-Npas4-ChIP-Seq(GSE127793)/Homer | 1e-4 | -1.108e+01 | 0.0000 | 1870.0 | 11.87% | 3661.9 | 10.83% | motif file (matrix) | svg |
| 175 | A C T G C A G T A C G T C G T A C G T A A C G T A C T G C T G A | Nkx6.1(Homeobox)/Islet-Nkx6.1-ChIP-Seq(GSE40975)/Homer | 1e-4 | -1.102e+01 | 0.0000 | 4535.0 | 28.80% | 9236.8 | 27.31% | motif file (matrix) | svg |
| 176 | G A C T C T A G C T A G C T A G A C T G T C G A C T G A C T A G C T A G C T A G G T A C G T C A | ZNF467(Zf)/HEK293-ZNF467.GFP-ChIP-Seq(GSE58341)/Homer | 1e-4 | -1.094e+01 | 0.0000 | 2650.0 | 16.83% | 5281.3 | 15.62% | motif file (matrix) | svg |
| 177 | G A C T C A G T G A T C G A T C A C G T G A T C C T G A T A C G C G T A G T C A | STAT6(Stat)/Macrophage-Stat6-ChIP-Seq(GSE38377)/Homer | 1e-4 | -1.089e+01 | 0.0000 | 1117.0 | 7.09% | 2123.3 | 6.28% | motif file (matrix) | svg |
| 178 | T A G C G A T C G T A C C T G A G C A T C T G A C G T A T G C A C G T A G A T C | Hoxa13(Homeobox)/ChickenMSG-Hoxa13.Flag-ChIP-Seq(GSE86088)/Homer | 1e-4 | -1.080e+01 | 0.0001 | 4527.0 | 28.74% | 9225.9 | 27.28% | motif file (matrix) | svg |
| 179 | T G C A G C A T A G C T G C A T A G T C A G T C A G T C C T G A A C T G T C G A T C G A C A G T A T C G A G T C G A T C | ZNF143\|STAF(Zf)/CUTLL-ZNF143-ChIP-Seq(GSE29600)/Homer | 1e-4 | -1.038e+01 | 0.0001 | 1022.0 | 6.49% | 1938.4 | 5.73% | motif file (matrix) | svg |
| 180 | T G C A C G T A G T C A A G C T A G T C G C T A T A G C C G A T C T A G G A T C | Gfi1b(Zf)/HPC7-Gfi1b-ChIP-Seq(GSE22178)/Homer | 1e-4 | -1.024e+01 | 0.0001 | 1414.0 | 8.98% | 2739.4 | 8.10% | motif file (matrix) | svg |
| 181 | A T G C A G T C C T G A A G T C C G A T A C G T A G T C A G T C A C G T A T C G G A C T A C G T | Etv2(ETS)/ES-ER71-ChIP-Seq(GSE59402)/Homer | 1e-4 | -1.015e+01 | 0.0001 | 2322.0 | 14.74% | 4616.9 | 13.65% | motif file (matrix) | svg |
| 182 | A G T C G A C T A G C T C G A T A T C G G C T A C G A T A T C G C G A T A C T G T A C G A C G T | Tcf7(HMG)/GM12878-TCF7-ChIP-Seq(Encode)/Homer | 1e-4 | -9.758e+00 | 0.0001 | 704.0 | 4.47% | 1306.9 | 3.86% | motif file (matrix) | svg |
| 183 | T C A G T A C G G A T C G T A C T C G A C G A T C T G A C G T A G C T A C G T A | Hoxd11(Homeobox)/ChickenMSG-Hoxd11.Flag-ChIP-Seq(GSE86088)/Homer | 1e-4 | -9.730e+00 | 0.0001 | 4470.0 | 28.38% | 9135.3 | 27.01% | motif file (matrix) | svg |
| 184 | G T A C C T A G T C A G A G C T T A G C C G T A A T G C T A C G A G T C G T A C G T C A A G T C | Srebp2(bHLH)/HepG2-Srebp2-ChIP-Seq(GSE31477)/Homer | 1e-4 | -9.682e+00 | 0.0001 | 315.0 | 2.00% | 541.4 | 1.60% | motif file (matrix) | svg |
| 185 | C G T A C T G A A C T G A G C T A G T C G A T C G A T C G C A T C T G A C T A G C T A G T A C G T C G A T G C A G C A T | EBF2(EBF)/BrownAdipose-EBF2-ChIP-Seq(GSE97114)/Homer | 1e-4 | -9.515e+00 | 0.0002 | 2246.0 | 14.26% | 4472.9 | 13.23% | motif file (matrix) | svg |
| 186 | A C T G G A C T A G T C C T G A G A T C T C A G A T G C G A C T A G T C A T G C T A G C A G C T A T C G T G C A | PAX5(Paired,Homeobox),condensed/GM12878-PAX5-ChIP-Seq(GSE32465)/Homer | 1e-4 | -9.497e+00 | 0.0002 | 296.0 | 1.88% | 506.3 | 1.50% | motif file (matrix) | svg |
| 187 | T C A G A G C T A C G T A C G T G T A C G A T C C G T A C T A G C A T G G T C A C G T A T C G A | STAT4(Stat)/CD4-Stat4-ChIP-Seq(GSE22104)/Homer | 1e-4 | -9.414e+00 | 0.0002 | 1927.0 | 12.24% | 3812.9 | 11.27% | motif file (matrix) | svg |
| 188 | C A T G T A C G T A G C G A T C G A T C A T G C G T A C G A C T T C A G A T G C C G A T A T C G C A G T A C T G G T A C | Zic3(Zf)/mES-Zic3-ChIP-Seq(GSE37889)/Homer | 1e-4 | -9.322e+00 | 0.0002 | 1800.0 | 11.43% | 3551.7 | 10.50% | motif file (matrix) | svg |
| 189 | T C G A A G T C C G T A A T C G T A G C A C G T A C T G A G C T A C G T A G T C | Ptf1a(bHLH)/Panc1-Ptf1a-ChIP-Seq(GSE47459)/Homer | 1e-4 | -9.315e+00 | 0.0002 | 6538.0 | 41.51% | 13543.2 | 40.05% | motif file (matrix) | svg |
| 190 | C G T A A C G T A C G T A C G T A C G T A G T C A G T C C T G A A G C T A G C T | NFAT(RHD)/Jurkat-NFATC1-ChIP-Seq(Jolma\_et\_al.)/Homer | 1e-3 | -9.185e+00 | 0.0002 | 1744.0 | 11.07% | 3438.1 | 10.17% | motif file (matrix) | svg |
| 191 | C G T A A C G T A C T G G T A C C G T A A C G T C G T A C G T A A C G T A C T G A G T C C G T A A C G T C T G A G C A T | OCT:OCT-short(POU,Homeobox)/NPC-OCT6-ChIP-Seq(GSE43916)/Homer | 1e-3 | -8.934e+00 | 0.0003 | 1101.0 | 6.99% | 2121.6 | 6.27% | motif file (matrix) | svg |
| 192 | A T G C T C G A A G T C A G C T A C G T G T A C A G T C G C T A C T A G C A T G G T C A C T G A T C A G A G T C | Stat3+il21(Stat)/CD4-Stat3-ChIP-Seq(GSE19198)/Homer | 1e-3 | -8.841e+00 | 0.0003 | 1593.0 | 10.11% | 3133.9 | 9.27% | motif file (matrix) | svg |
| 193 | C A G T C G A T G C A T G C A T G T C A A G C T C A T G C T A G A T G C G T A C | Hoxa11(Homeobox)/ChickenMSG-Hoxa11.Flag-ChIP-Seq(GSE86088)/Homer | 1e-3 | -8.826e+00 | 0.0003 | 4261.0 | 27.06% | 8720.6 | 25.79% | motif file (matrix) | svg |
| 194 | C G T A C T G A C G T A C T A G T C G A C T A G A C T G C G T A C G T A T A C G A G C T A T C G | SpiB(ETS)/OCILY3-SPIB-ChIP-Seq(GSE56857)/Homer | 1e-3 | -8.678e+00 | 0.0004 | 518.0 | 3.29% | 948.4 | 2.80% | motif file (matrix) | svg |
| 195 | T C A G G T C A G C T A A G T C C G T A A T C G T C G A T C A G C G T A A C T G A C T G A C T G | ZNF768(Zf)/Rajj-ZNF768-ChIP-Seq(GSE111879)/Homer | 1e-3 | -8.266e+00 | 0.0006 | 96.0 | 0.61% | 142.0 | 0.42% | motif file (matrix) | svg |
| 196 | T G A C A T G C C G T A A T C G A T G C C A G T C A T G A C T G A G T C G T A C | HEB(bHLH)/mES-Heb-ChIP-Seq(GSE53233)/Homer | 1e-3 | -8.204e+00 | 0.0006 | 4955.0 | 31.46% | 10210.6 | 30.19% | motif file (matrix) | svg |
| 197 | G C T A A C G T C T A G G T C A C G T A A C G T C G T A C G A T C A G T A G T C C G T A A C G T C T A G C T G A A T C G | OCT:OCT(POU,Homeobox)/NPC-Brn1-ChIP-Seq(GSE35496)/Homer | 1e-3 | -8.195e+00 | 0.0006 | 22.0 | 0.14% | 20.8 | 0.06% | motif file (matrix) | svg |
| 198 | C T A G T A G C A G T C G T C A C T G A A C G T C G T A C G T A C G T A G C T A | Hoxd13(Homeobox)/ChickenMSG-Hoxd13.Flag-ChIP-Seq(GSE86088)/Homer | 1e-3 | -7.566e+00 | 0.0012 | 2842.0 | 18.05% | 5767.9 | 17.06% | motif file (matrix) | svg |
| 199 | C G A T G A C T C G A T T C A G G A C T A C G T C A G T C T G A G A C T G A C T A G C T C G A T A C T G A T C G G T A C G C T A | NF1:FOXA1(CTF,Forkhead)/LNCAP-FOXA1-ChIP-Seq(GSE27824)/Homer | 1e-3 | -7.536e+00 | 0.0012 | 113.0 | 0.72% | 175.6 | 0.52% | motif file (matrix) | svg |
| 200 | T G A C G T C A A G T C G C T A C T A G A G T C C T G A C A T G A C T G C T A G T A C G T C A G | Zic2(Zf)/ESC-Zic2-ChIP-Seq(SRP197560)/Homer | 1e-3 | -7.458e+00 | 0.0013 | 1422.0 | 9.03% | 2808.6 | 8.30% | motif file (matrix) | svg |
| 201 | G C A T C T A G G T A C A G T C C G A T A C T G C T A G C T A G G T A C G C T A | ZNF416(Zf)/HEK293-ZNF416.GFP-ChIP-Seq(GSE58341)/Homer | 1e-3 | -7.444e+00 | 0.0013 | 3532.0 | 22.43% | 7223.5 | 21.36% | motif file (matrix) | svg |
| 202 | C T G A A C G T A C G T A C G T A G T C G A C T C G A T C T G A A C T G C G T A C G T A T C G A | STAT5(Stat)/mCD4+-Stat5-ChIP-Seq(GSE12346)/Homer | 1e-3 | -7.432e+00 | 0.0013 | 732.0 | 4.65% | 1394.1 | 4.12% | motif file (matrix) | svg |
| 203 | T A C G T A C G C T A G T C A G A G T C C G T A A T C G A T G C A C G T A C T G A G T C G A C T | Ascl2(bHLH)/ESC-Ascl2-ChIP-Seq(GSE97712)/Homer | 1e-3 | -7.288e+00 | 0.0015 | 3105.0 | 19.72% | 6328.8 | 18.71% | motif file (matrix) | svg |
| 204 | T A C G T C A G T G C A A G C T T G A C A G C T A G T C A C T G G A T C A C T G T C G A A C T G C T G A C T G A A T G C | ZBTB33(Zf)/GM12878-ZBTB33-ChIP-Seq(GSE32465)/Homer | 1e-3 | -7.265e+00 | 0.0015 | 194.0 | 1.23% | 328.9 | 0.97% | motif file (matrix) | svg |
| 205 | C T A G A T C G G A C T A G T C A G T C A G T C G A C T C T G A A C T G C T A G A C T G C T G A | EBF(EBF)/proBcell-EBF-ChIP-Seq(GSE21978)/Homer | 1e-3 | -7.189e+00 | 0.0016 | 543.0 | 3.45% | 1016.7 | 3.01% | motif file (matrix) | svg |
| 206 | A C T G C A T G G C T A T C G A G C T A A G C T A G C T G T A C A G T C T G A C | NFkB-p65-Rel(RHD)/ThioMac-LPS-Expression(GSE23622)/Homer | 1e-2 | -6.802e+00 | 0.0024 | 119.0 | 0.76% | 190.6 | 0.56% | motif file (matrix) | svg |
| 207 | T G A C G T A C C G T A A C T G T G A C C G A T A C T G A T C G A G C T T A C G T C G A T A G C G T A C C G T A A T C G T G A C G C A T A C T G A C T G A T G C | Twist(bHLH)/HMLE-TWIST1-ChIP-Seq(Chang\_et\_al)/Homer | 1e-2 | -6.695e+00 | 0.0026 | 345.0 | 2.19% | 627.5 | 1.86% | motif file (matrix) | svg |
| 208 | C T G A A T G C C G T A A C G T A G T C A G T C A C G T A C T G A T C G G C A T | SPDEF(ETS)/VCaP-SPDEF-ChIP-Seq(SRA014231)/Homer | 1e-2 | -6.449e+00 | 0.0033 | 2467.0 | 15.66% | 5012.3 | 14.82% | motif file (matrix) | svg |
| 209 | T G A C T C G A C T G A C T G A A T G C G A T C C T A G T A C G G A C T G A C T G A T C T C G A C T G A C T G A A T G C G A T C C T A G A T C G G A C T G A C T | Tcfcp2l1(CP2)/mES-Tcfcp2l1-ChIP-Seq(GSE11431)/Homer | 1e-2 | -6.371e+00 | 0.0036 | 389.0 | 2.47% | 718.4 | 2.12% | motif file (matrix) | svg |
| 210 | A T C G A G C T C T G A C T A G A C T G A C G T G T A C G C T A A T G C A C G T C T A G C A T G T A C G C G A T A T G C C G T A | Reverb(NR),DR2/RAW-Reverba.biotin-ChIP-Seq(GSE45914)/Homer | 1e-2 | -6.091e+00 | 0.0047 | 322.0 | 2.04% | 588.4 | 1.74% | motif file (matrix) | svg |
| 211 | A T G C G A T C G A C T A G C T C G A T C G A T G T C A C G A T T C G A A T C G T A G C T A G C | TATA-Box(TBP)/Promoter/Homer | 1e-2 | -6.059e+00 | 0.0049 | 2500.0 | 15.87% | 5093.9 | 15.06% | motif file (matrix) | svg |
| 212 | A G T C C G A T C T G A C G T A A C G T C A G T T C A G T G A C | Isl1(Homeobox)/Neuron-Isl1-ChIP-Seq(GSE31456)/Homer | 1e-2 | -5.932e+00 | 0.0055 | 3658.0 | 23.23% | 7540.0 | 22.30% | motif file (matrix) | svg |
| 213 | T A G C T C A G C A T G G C A T A G C T C G A T A T G C C G T A C G T A G T C A | CHR(?)/Hela-CellCycle-Expression/Homer | 1e-2 | -5.865e+00 | 0.0059 | 1053.0 | 6.69% | 2079.9 | 6.15% | motif file (matrix) | svg |
| 214 | C A T G A C G T A G T C G A T C G A T C G A T C G C T A C T A G C T A G C T A G T C A G T C G A | EBF1(EBF)/Near-E2A-ChIP-Seq(GSE21512)/Homer | 1e-2 | -5.795e+00 | 0.0063 | 2756.0 | 17.50% | 5641.0 | 16.68% | motif file (matrix) | svg |
| 215 | A G T C G T A C A G C T C T A G A G T C C G A T A C T G C G T A A C T G G T C A | Zic(Zf)/Cerebellum-ZIC1.2-ChIP-Seq(GSE60731)/Homer | 1e-2 | -5.548e+00 | 0.0080 | 2225.0 | 14.13% | 4531.1 | 13.40% | motif file (matrix) | svg |
| 216 | C G T A A C G T A G C T C G A T C T A G G T A C C G T A A G C T C G T A G C T A | Oct4(POU,Homeobox)/mES-Oct4-ChIP-Seq(GSE11431)/Homer | 1e-2 | -5.473e+00 | 0.0086 | 806.0 | 5.12% | 1578.1 | 4.67% | motif file (matrix) | svg |
| 217 | G A C T G T A C T G C A A C G T G A T C G C T A T C G A A C G T A G T C C G T A | Pdx1(Homeobox)/Islet-Pdx1-ChIP-Seq(SRA008281)/Homer | 1e-2 | -5.420e+00 | 0.0090 | 1636.0 | 10.39% | 3301.3 | 9.76% | motif file (matrix) | svg |
| 218 | T C A G T C A G G C T A C G T A T A C G G A C T T C A G T C G A C T G A C G T A T A C G G A C T | IRF8(IRF)/BMDM-IRF8-ChIP-Seq(GSE77884)/Homer | 1e-2 | -5.381e+00 | 0.0093 | 674.0 | 4.28% | 1309.7 | 3.87% | motif file (matrix) | svg |
| 219 | C T G A C G A T C T A G C G T A A G C T C G A T C A G T C T G A G A C T C T A G C T A G A T G C | PBX2(Homeobox)/K562-PBX2-ChIP-Seq(Encode)/Homer | 1e-2 | -5.042e+00 | 0.0130 | 1440.0 | 9.14% | 2902.6 | 8.58% | motif file (matrix) | svg |
| 220 | C T G A A G C T A C G T A C G T A G T C G A C T G A C T C T G A C T G A C T A G C G T A C G T A | STAT6(Stat)/CD4-Stat6-ChIP-Seq(GSE22104)/Homer | 1e-2 | -5.029e+00 | 0.0131 | 1008.0 | 6.40% | 2004.2 | 5.93% | motif file (matrix) | svg |
| 221 | C A T G T G A C C G T A A G T C T A C G G C A T A C T G G T C A A T G C A G T C | bHLHE41(bHLH)/proB-Bhlhe41-ChIP-Seq(GSE93764)/Homer | 1e-2 | -4.990e+00 | 0.0136 | 2939.0 | 18.66% | 6054.1 | 17.90% | motif file (matrix) | svg |
| 222 | G A C T T C G A C G A T T A C G C T A G T A C G A C T G A T G C G T A C G T A C | Zac1(Zf)/Neuro2A-Plagl1-ChIP-Seq(GSE75942)/Homer | 1e-2 | -4.980e+00 | 0.0136 | 6744.0 | 42.82% | 14153.0 | 41.85% | motif file (matrix) | svg |
| 223 | T G A C C T A G A C T G T A G C C G A T A C T G A T G C C A T G A T C G A T C G A T C G T A G C C T G A T A G C G C T A A C T G C G T A A G C T C G T A C T G A | GATA:SCL(Zf,bHLH)/Ter119-SCL-ChIP-Seq(GSE18720)/Homer | 1e-2 | -4.946e+00 | 0.0140 | 235.0 | 1.49% | 428.4 | 1.27% | motif file (matrix) | svg |
| 224 | G T C A C T G A T C A G G A T C T G C A T G C A A C G T T C A G C G T A C G T A C G T A G C T A | Hoxd12(Homeobox)/ChickenMSG-Hoxd12.Flag-ChIP-Seq(GSE86088)/Homer | 1e-2 | -4.858e+00 | 0.0153 | 3244.0 | 20.60% | 6704.3 | 19.82% | motif file (matrix) | svg |
| 225 | C T A G C A T G A C G T A G T C G C T A A G C T A G T C A G C T T C A G C T G A A C T G C A T G G C A T A T G C C G T A | THRa(NR)/C17.2-THRa-ChIP-Seq(GSE38347)/Homer | 1e-2 | -4.752e+00 | 0.0169 | 949.0 | 6.03% | 1888.9 | 5.59% | motif file (matrix) | svg |
| 226 | T C G A C A T G C A T G T A C G G T A C T C G A A G T C C A G T C T G A C G T A A G T C G A C T | ZNF264(Zf)/HEK293-ZNF264.GFP-ChIP-Seq(GSE58341)/Homer | 1e-2 | -4.689e+00 | 0.0179 | 1565.0 | 9.94% | 3173.9 | 9.38% | motif file (matrix) | svg |
